# Characteristics of the Energetic Igniters Through Integrating B/Ti Nano-Multilayers on TaN Film Bridge

**DOI:** 10.1186/s11671-015-0934-z

**Published:** 2015-05-29

**Authors:** YiChao Yan, Wei Shi, HongChuan Jiang, XianYao Cai, XinWu Deng, Jie Xiong, WanLi Zhang

**Affiliations:** State Key Laboratory of Electronic Thin Films and Integrated Devices, University of Electronic Science and Technology of China, Chengdu, 610054 China

**Keywords:** TaN film bridge, (B/Ti)_*n*_/TaN, Temperature coefficient of resistance, Electrical explosion

## Abstract

The energetic igniters through integrating B/Ti nano-multilayers on tantalum nitride (TaN) ignition bridge are designed and fabricated. The X-ray diffraction (XRD) and temperature coefficient of resistance (TCR) results show that nitrogen content has a great influence on the crystalline structure and TCR. TaN films under nitrogen ratio of 0.99 % exhibit a near-zero TCR value of approximately 10 ppm/°C. The scanning electron microscopy demonstrates that the layered structure of the B/Ti multilayer films is clearly visible with sharp and smooth interfaces. The electrical explosion characteristics employing a capacitor discharge firing set at the optimized charging voltage of 45 V reveal an excellent explosion performance by (B/Ti)_*n*_/TaN integration film bridge with small ignition delay time, high explosion temperature, much more bright flash of light, and much large quantities of the ejected product particles than TaN film bridge.

## Background

With the increasing demand for small ignition devices, the investigation of heavily semiconductor or metallic film bridge, especially doped polycrystalline silicon, titanium, platinum, and chromium bridge, has attracted much attention in recent years [1–7]. Film ignition bridge devices, which are over 30 times smaller in volume, can function in a few tens of microseconds and operate at one-tenth the input energy compared with the hot-wire devices while improving no-fire conditions and electrostatic safety. In the discharge mechanism of film ignition bridge, thermal plasma is generated to ignite explosive powder by passing current through the bridge which in turn changes the physical features of the bridge. On the ignition bridge, a variety of reactive multilayer films which consist of alternating nanoscale layers of metal or metal oxide such as Al/Ni, B/Ti, Al/CuO, and Al/MoO_*x*_ [8–16] are deposited to provide large negative reaction heats. A small thermal pulse or an electrical stimulus along the films causes atoms to diffuse normal to the layers and results in a rapid self-propagating exothermic reaction. The integrated structure combines the advantages of film ignition bridge devices and reactive multilayer films, which may improve the ignition performance and reliability in the case of low electrical energy consumption, fast energy release rate, and large amount of reaction heat.

Tantalum nitride (TaN) can be a promising candidate for high-precision thin-film resistor and its excellent physical properties including quite high values of sheet resistance [17]. The temperature coefficient of resistance (TCR) of the film is adjusted by changing the sputtering parameters, which makes it ideally suitable for the application across a large temperature range compared with traditional metallic film bridge devices.

In this paper, an energetic initiator through integrating the B/Ti reactive multilayer films with tantalum nitride film bridge is designed and fabricated. The effects of nitrogen content on the crystalline structure and temperature coefficient of resistance of TaN are systematically investigated. The electrical explosion properties and ignition flame temperature of TaN film and (B/Ti)_*n*_/TaN integration film ignition bridge are comparatively studied.

## Methods

**Fig. 1:**
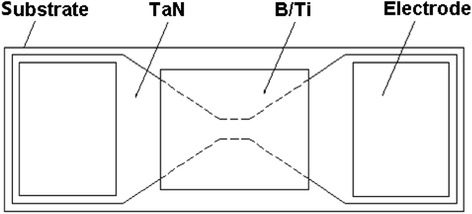
Schematic view of an initiator by integrating the B/Ti reactive multilayer films with tantalum nitride film bridge

A schematic diagram of the experimental (B/Ti)_*n*_/TaN integrated device is shown in Fig. 1, which consists of an “H” shaped TaN polycrystalline film with wet-etching, subsequent square shape (B/Ti)_*n*_ multilayers with mask of 4 × 4 mm, and two lands of copper electrode. The dimensions of the bridge are 80 μm long (*l*) by 40 μm wide (*w*) by 2 μm thick (*t*). The parameters *l* and *w* would influence the energy loss into the substrate, and the two metal lands are designed to maximize the metal/TaN contact area to insure much less contact resistance.

TaN films are deposited onto alumina substrates (10 × 5 × 0.5 mm) by direct current (DC) reactive magnetron sputtering. Before deposition, the substrates are cleaned with acetone, alcohol, and de-ionized water in an ultrasonic bath for 10 min, respectively, which subsequently are dried by nitrogen gas and annealed in the oven at 200 °C for 2 h. A tantalum cylinder (99.995 % purity) with a diameter of 60 mm is applied for the sputtering target, and the distance between target and substrate is 80 mm for consideration to provide the best deposition rate and uniformity of thin films. When the base pressure is pumped down to 5 × 10^−4^ Pa, argon gas is firstly introduced into the chamber as the work gas, and then, a thin tantalum metal film is deposited for 20 min as the interlayer. Nitrogen (99.999 %) gas is introduced into the chamber as reactive gas. To determine the effect of nitrogen partial pressure, the percentage of N_2_/(N_2_ + Ar) gas ratio is varied from 0.33 to 2 %. TaN layers are deposited for 30 min with sputtering temperature, pressure, and power of 200 °C, 0.33 Pa, and 120 W, respectively. After the deposition, reversal photoresist (PRI-9000A) is spin-coated onto the TaN film and patterned using photolithography through a designed mask. Once the photoresist is exposed, TaN film is rinsed in the developer and then placed in the oven of 100 °C for 1 h. Subsequently, the exposed TaN film is directly wet-etched in the corrosive liquid.

B/Ti multilayer films are sputter deposited from B(99.995 % purity) and Ti(99.995 % purity) targets with the diameter of 100 mm by radio frequency (RF) and DC magnetron sputtering, respectively. The substrates move alternately under the afterglow from sputtered B and the Ti target through a shield with optimized shape to allow for homogeneous film atoms. The base pressure is below 5 × 10^−4^ Pa, and the substrate temperature remains at 100 °С in order to prevent the interdiffusion and reaction during deposition. The sputtering power is fixed at 200 and 160 W for B and Ti, respectively. The thickness ratio of B to Ti per period is maintained at 1:1 to obtain the multilayer films with an average stoichiometric ratio of TiB_2_. The total thickness of B/Ti multilayer films is 8 μm, and the modulation period thickness is 400 nm.

The crystallographic structure of TaN films is determined by Bede D1 X-ray diffraction using Cu Ka radiation. Film resistance is calculated from the sheet resistance measured by a SDY-4 four-point probe, and the film thickness is measured by a Dektak150 profilometer. The cross-sectional morphologies of multilayer films are characterized using scanning electron microscopy (SEM).

The principle of open-air electrical explosion testing of the initiator is introduced in detail in a few literatures [18–20]. The parameters including the ignition voltage, ignition current, and ignition delay are achieved by using capacitor voltage discharging firing set with and without the presence of the B/Ti reactive multilayer films. The explosion temperature of the initiator is determined based on the “double-line atomic emission spectroscopy of a copper element” with the fundamental principle described in this literature [18, 19]. A high-speed camera (HS4540MX12) with 20,000 frames per second resolution is used to observe the explosion process directly.

## Results and Discussion

**Fig. 2:**
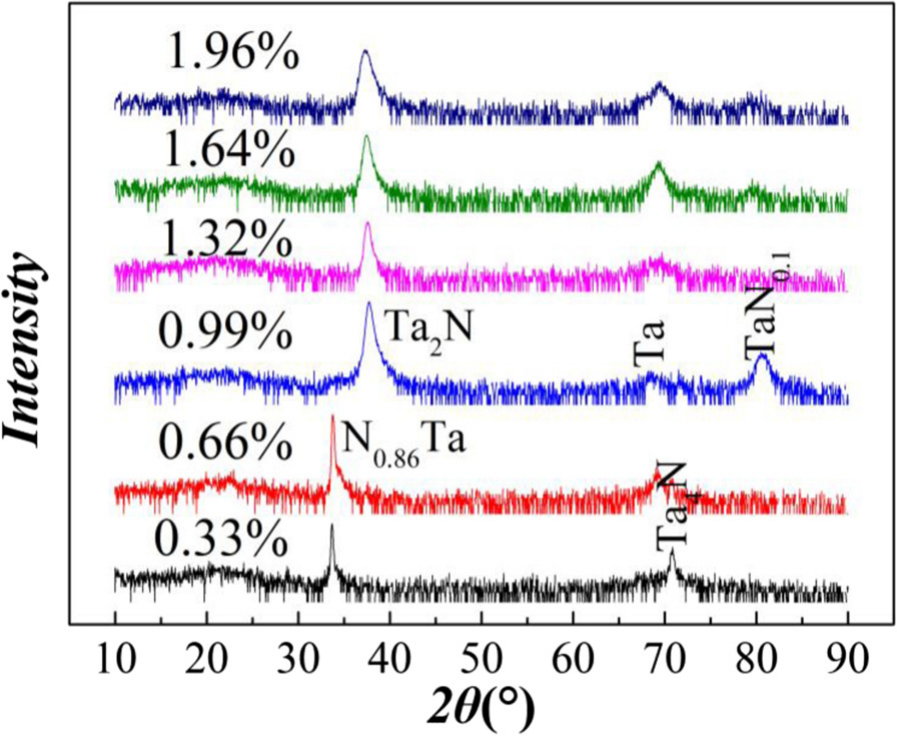
XRD spectra of TaN films deposited on the alumina substrate with various ratios of N_2_/(N_2_ + Ar)

**Fig. 3:**
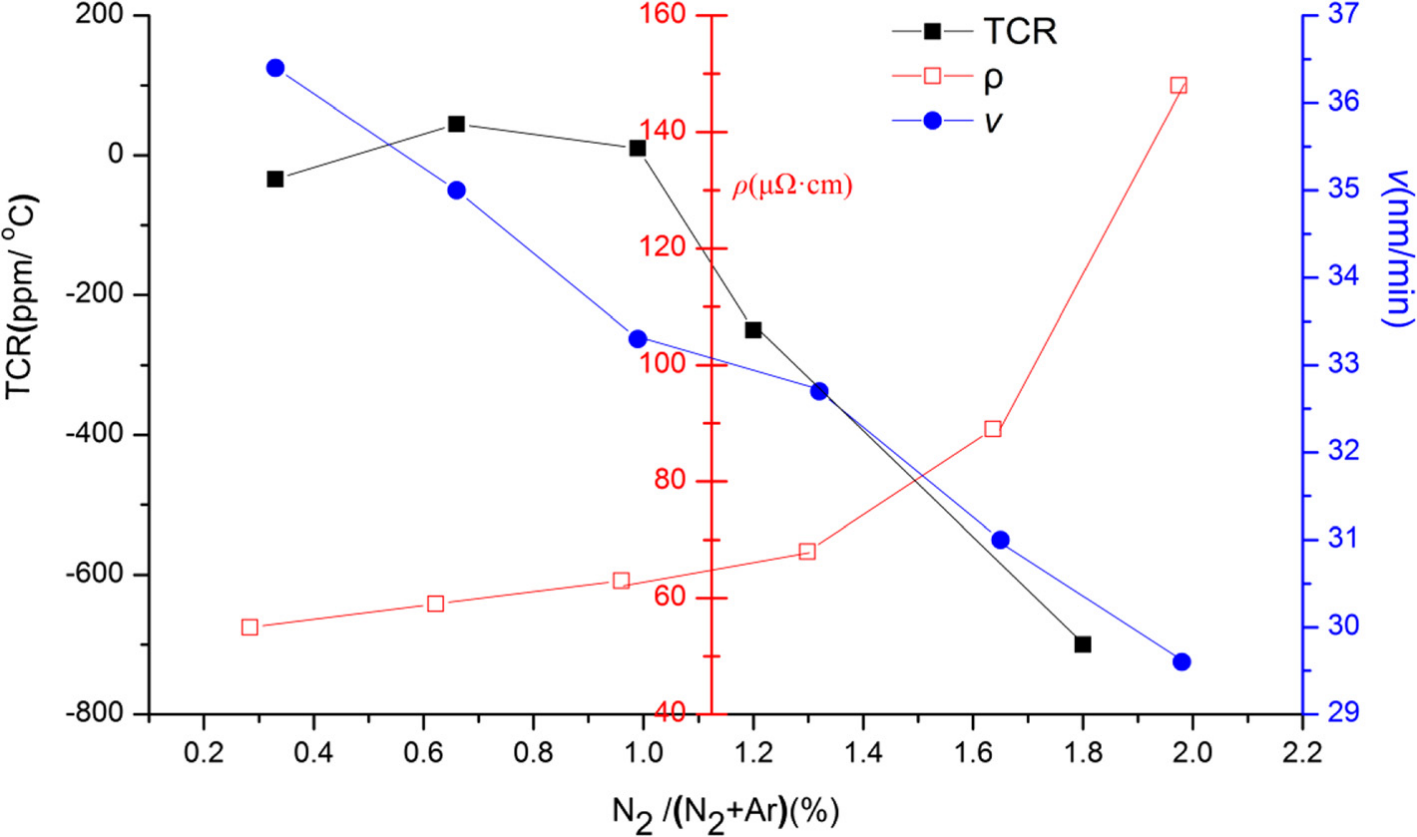
The resistivity, deposition rate, and temperature coefficient of resistance of TaN films as a function of nitrogen ratio

The X-ray diffraction (XRD) spectra of 2-μm-thick TaN films on alumina substrate with various nitrogen ratio are shown in Fig. 2. As the nitrogen ratio increases, the main phases of the films evolve from Ta to Ta_*x*_N. At low N_2_/(N_2_ + Ar) gas ratio from 0.33 to 0.66 %, only TaN_0.86_ and Ta phase are observed. With the increase of the nitrogen gas ratio from 0.66 to 1.96 %, the films are preferentially formed Ta_2_N phase with TaN_0.1_ phase and remanent Ta. Although N-rich phases are not present in the XRD spectra, the increased resistivity of TaN films as a function of the nitrogen ratio indicates the existence of N-rich phase in Fig. 3. A gradual increase in resistivity of TaN films with nitrogen ratio ranging from 0.33 to 1.3 % is observed; however, over the percentage of 1.3 %, an abrupt increase emerges. The deposition rate (*ν*) decreases almost linearly with an increase of nitrogen ratio, which is attributed to Ta meal target poisoning, and further demonstrates the N-rich environment.

The temperature coefficient of resistance of TaN films decreases with an increase of nitrogen ratio, as shown in Fig. 3. The difference in TCR as a function of nitrogen ratio is mainly affected by the phases in the films. The near-zero TCR corresponds to the existence of TaN_0.86_ when the nitrogen ratio is below 1 %; however, the appearance of Ta_2_N phase leads to much larger value of negative TCR with the nitrogen ratio shifting from 1 to 1.96 %. Due to the presence of Ta phase as shown in the XRD spectra, a further reduced TCR value of TaN film could be achieved, because the positive TCR value of the Ta phase can counteract the negative part of Ta_2_N phase. For our film ignition bridges, the resistance of the films are required to be controlled between 1 and 4 Ω, and hence, much smaller and even near-zero TCR in TaN films with low resistivity is favorable for the properties of the devices. Above all, the nitrogen ratio of 0.99 % is preferable to grow TaN film, exhibiting a near-zero TCR of approximately 10 ppm/°С.

**Fig. 4:**
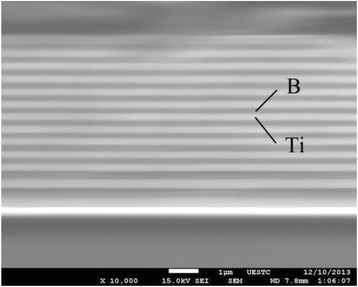
The cross-sectional SEM morphology of the B/Ti multilayer films

Figure 4 shows the cross-sectional morphology of the B/Ti multilayer films. The layered structure of the B/Ti multilayer films is clearly visible with sharp and smooth interfaces.

**Fig. 5:**
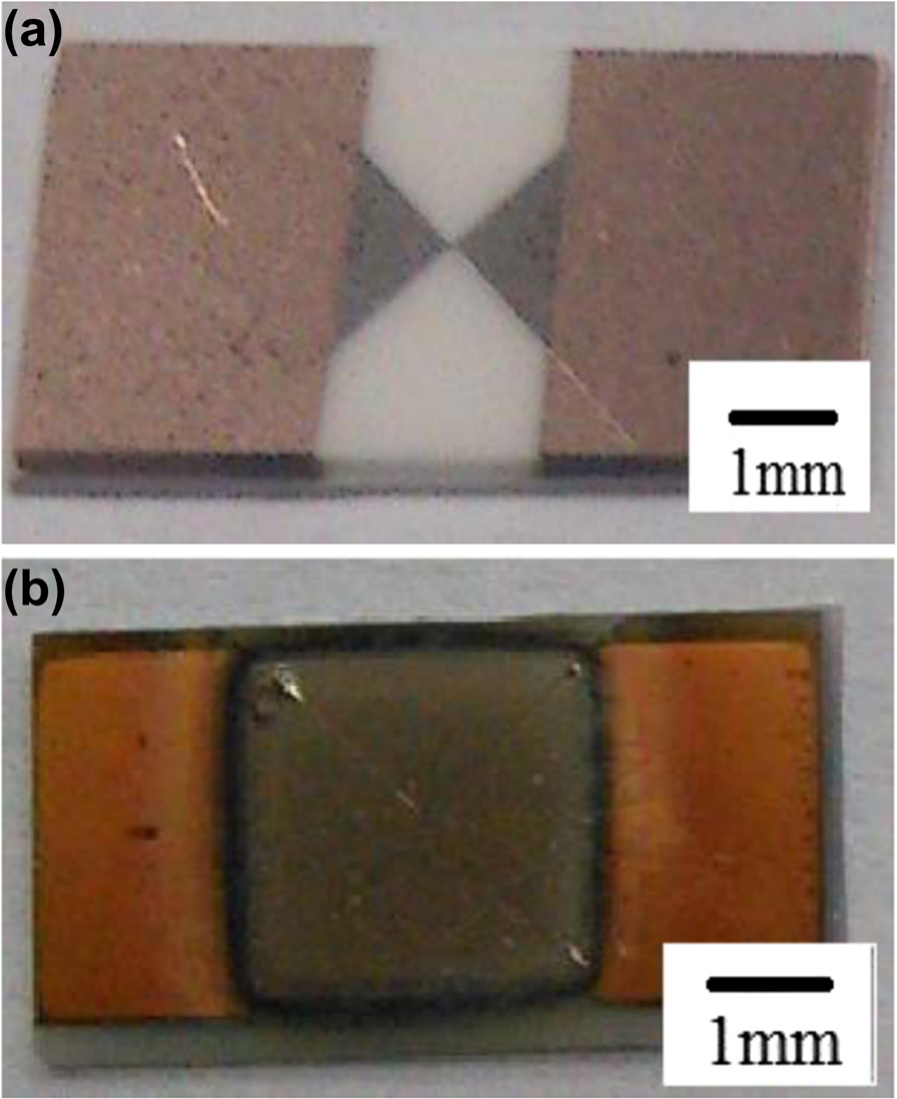
Microscopic image of TaN film bridge (**a**) and (B/Ti)_*n*_/TaN integration film bridge (**b**)

For the evaluation of electrical explosion behaviors of TaN and (B/Ti)_*n*_/TaN film bridge, a capacitor discharge firing circuit (47 μF, 45 V, or 25 V) is adopted to apply the currents across the film bridge with an electrical trigger pulse. The electrical explosion process is totally different for TaN and (B/Ti)_*n*_/TaN film bridge, and thus, a corresponding optimized discharging voltage for a specific structure of film bridge in Fig. 5 is necessary.

**Fig. 6:**
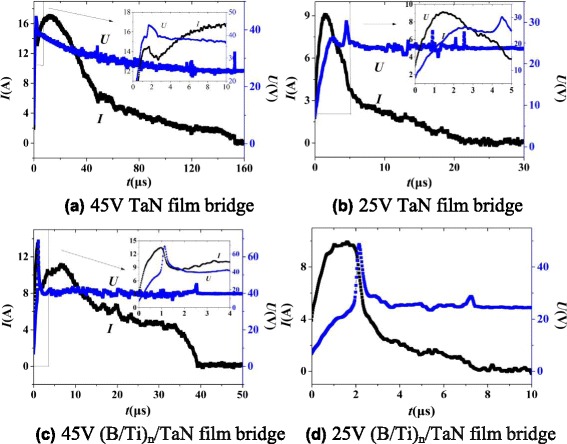
Voltage-current histories for TaN film and (B/Ti)_*n*_/TaN film bridge. **a** 45-V TaN film bridge. **b** 25-V TaN film bridge. **c** 45-V (B/Ti)_*n*_/TaN film bridge. **d** 25-V (B/Ti)_*n*_/TaN film bridge, *insets* show partial enlarged details of the selected areas

Figure 6 shows the variation of the voltage and current as a function of time for TaN film and (B/Ti)_*n*_/TaN film bridge with the capacitor voltage charged to 45 and 25 V, respectively. Due to the existence of 400-nm-thick B interlayer as a insulation layer between the TaN film and B/Ti nano-multilayers, current should pass through the bottom layer of TaN film for (B/Ti)_*n*_/TaN film bridge, so voltage-current curves for (B/Ti)_*n*_/TaN film bridge are similar with those of TaN film bridge. By the comparison of voltage-current curves of TaN film and (B/Ti)_*n*_/TaN film bridge, 45 V is considered as the optimized charging voltage at which the voltage and current curves almost reach the peak simultaneously. The time of reaching the peak value of voltage for (B/Ti)_*n*_/TaN film bridge at 45 V is almost half as that of TaN film bridge, meaning the rapid melting, vaporization, and ionization process of (B/Ti)_*n*_/TaN film bridge because of the less energy loss to the open air. Meanwhile, the ignition duration time of (B/Ti)_*n*_/TaN film bridge is almost one fourth as that of TaN film bridge, which gives rise to more energy released in a short time.

**Fig. 7:**
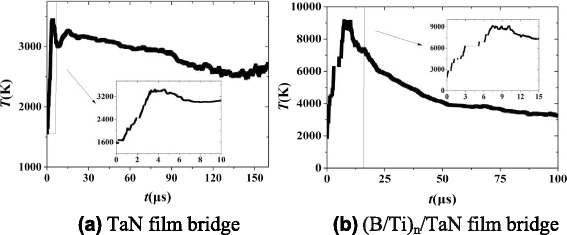
Explosion temperature histories vs. its duration time for TaN film (**a**) and (B/Ti)_*n*_/TaN film bridge (**b**), *insets* show partial enlarged details of the selected areas

**Fig. 8:**
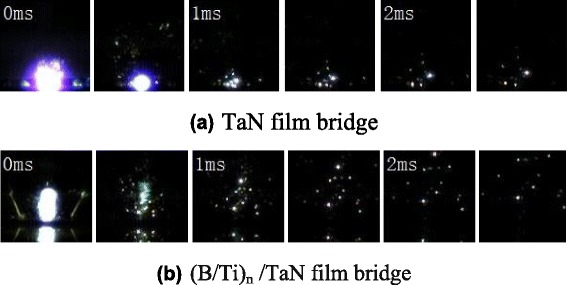
High-speed camera observation of electrical explosion process for TaN film (**a**) and (B/Ti)_*n*_/TaN film bridge (**b**)

Figure 7 shows the comparison of explosion temperature for TaN film and (B/Ti)_*n*_/TaN film bridge both charged to 45 V. Due to the large energy released through the intermetallic reaction of B/Ti multilayers, the peak explosion temperature is about 9000 K for (B/Ti)_*n*_/TaN film bridge which is almost three times than that of TaN film bridge. However, once the voltage supply is triggered, the time of reaching the peak value of explosion temperature for (B/Ti)_*n*_/TaN film bridge is about two times than that of TaN film bridge, which is ascribed to relatively slow reaction velocity for nano-multilayers compared with the ionization process of TaN film bridge. The explosion temperature histories are consistent with high-speed camera observation of electrical explosion process for TaN film and (B/Ti)_*n*_/TaN film bridge, as shown in Fig. 8. For (B/Ti)_*n*_/TaN film bridge, a more fierce explosion process is observed accompanied with much more bright flash of light, much large quantities, and longer distance of the ejected product particles by comparison with TaN film bridge, which could definitely conclude that the integration of B/Ti multilayers can improve the ignition performance obviously.

## Conclusions

The purpose of this work is to gain a better understanding of the electrical explosion characteristics of TaN film and (B/Ti)_*n*_/TaN film bridge ignition devices. From the electrical explosion characteristics comparing the TaN film and (B/Ti)_*n*_/TaN film bridge, the peak voltage of (B/Ti)_*n*_/TaN film bridge precedes that of TaN film bridge at the time of plasma generation, and the corresponding peak temperature is almost three times than that of TaN film bridge, which is attributed to large energy released through the intermetallic reaction of B/Ti multilayers. High-speed camera reveals a more fierce combustion procedure for (B/Ti)_*n*_/TaN film bridge. In a word, the advantage of low input ignition energy, the adjustable temperature coefficient of resistance at any time, smaller response time, and high-temperature ejected product particles make the (B/Ti)_*n*_/TaN film bridge superior to the TaN film bridge ignition device and ignite the explosives directly without assisted explosive powders. It should be noted that (B/Ti)_*n*_/TaN film bridge ignition device could be realized by standard microfabrication techniques that allow batch fabrication and high level of integration.
